# Virstatin inhibits biofilm formation and motility of *Acinetobacter baumannii*

**DOI:** 10.1186/1471-2180-14-62

**Published:** 2014-03-12

**Authors:** Yassine Nait Chabane, Mohamed Ben Mlouka, Stéphane Alexandre, Marion Nicol, Sara Marti, Martine Pestel-Caron, Jordi Vila, Thierry Jouenne, Emmanuelle Dé

**Affiliations:** 1 Normandie University, Caen, France; 2 Laboratory “Polymères, Biopolymères, Surfaces”, UMR 6270 & FR 3038 CNRS, IRIB, University of Rouen, Mont-Saint-Aignan, Cedex 76821, France; 3 University of Rouen, Rouen University Hospital, GRAM, Rouen, EA 2656, France; 4 Department of Microbiology, Hospital Clinic, Barcelona, Spain

**Keywords:** Pellicle, Protein-protein interactions, Type IV pili, Virstatin

## Abstract

**Background:**

*Acinetobacter baumannii* has emerged as an opportunistic nosocomial pathogen causing infections worldwide. One reason for this emergence is due to its natural ability to survive in the hospital environment, which may be explained by its capacity to form biofilms. Cell surface appendages are important determinants of the *A. baumannii* biofilm formation and as such constitute interesting targets to prevent the development of biofilm-related infections. A chemical agent called virstatin was recently described to impair the virulence of *Vibrio cholerae* by preventing the expression of its virulence factor, the toxin coregulated pilus (type IV pilus). The objective of this work was to investigate the potential effect of virstatin on *A. baumannii* biofilms*.*

**Results:**

After a dose–response experiment, we determined that 100 μM virstatin led to an important decrease (38%) of biofilms formed by *A. baumannii* ATCC17978 grown under static mode. We demonstrated that the production of biofilms grown under dynamic mode was also delayed and reduced. The biofilm susceptibility to virstatin was then tested for 40 clinical and reference *A. baumannii* strains. 70% of the strains were susceptible to virstatin (with a decrease of 10 to 65%) when biofilms grew in static mode, whereas 60% of strains respond to the treatment when their biofilms grew in dynamic mode. As expected, motility and atomic force microscopy experiments showed that virstatin acts on the *A. baumannii* pili biogenesis.

**Conclusions:**

By its action on pili biogenesis, virstatin demonstrated a very promising antibiofilm activity affecting more than 70% of the *A. baumannii* clinical isolates.

## Background

*Acinetobacter baumannii*, a microorganism with a worldwide epidemic spread, causes a wide range of infections, including pneumonia and blood-stream infections. This increasing threat in hospitals is mainly due to the occurrence of multidrug-resistant strains, associated with the real problem of eradication in the hospital wards [1]. Biofilm formation may facilitate the environmental survival of *A. baumannii* by conferring resistance to antibiotics, desiccation or nutritional stress and explain the success of particular strains in hospitals [2,3]. Several factors have been proved to play a role in this biofilm formation or maturation, like the poly-β-(1–6)-N-acetyl glucosamine extracellular polysaccharide, the biofilm-associated protein, the autotransporter Ata or the systems of protein glycosylation [4-7]. In addition, extracellular appendages are often involved in different stages of bacterial biofilm [8] as exemplified by the *csuA/BABCDE* chaperon-usher system, coding for fimbriae, and required for initial steps of *A. baumannii* biofilm development [6,9,10]. Recent findings have also demonstrated that more than one cell surface appendages system may be involved in the maintenance of the biofilm structure, especially when the biofilm is formed at the air-liquid interface [11]. Finally, the presence of a type IV pili system and its involvement in motility was recently described in *A. baumannii*[12]. It could also play a role in biofilm development (initial adhesion, microcolonies formation and in maturation, last step of biofilm formation) as demonstrated for other bacterial species [8,13]. Therefore, the contribution of extracellular appendages to the biofilm structuration makes them very attractive as therapeutic targets [14].

In this study, we experienced this new approach by testing the use of the chemical agent virstatin. This small organic molecule has been demonstrated to inhibit *Vibrio cholerae* virulence and its orogastric administration would protect infant mice from *V. cholerae* intestinal colonization [15,16]. Virstatin acted in preventing expression of the two major *Vibrio cholerae* virulence factors, cholera toxin and the toxin coregulated pilus (a type IV pilus, T4P). It would disrupt protein-protein interactions, *i.e.* the dimerization of the transcriptional regulator ToxT, stopping thus the activation of *ctx* and *tcp* genes [15,16].

We present here the efficacy of the virstatin as an inhibitor of the pili system synthesis to prevent *A. baumannii* biofilm formation.

## Methods

### Bacterial strains and MICs determination

We used *A. baumannii* ATCC 17978 and ATCC 19606 as reference strains, as well as 38 clonally unrelated *A. baumannii* clinical isolates, among which multidrug-resistant (MDR) and extensively drug-resistant (XDR) strains [2,17]. Nineteen clinical isolates formed biofilm on solid support and the remaining 19 isolates had the ability to form a pellicle [18]. Determination of MICs of virstatin (4-[*N*-(1,8-naphthalimide)]-*n*-butyric acid; Bachem, Weil am Rhein, Germany) solubilized in dimethyl sulfoxide (DMSO, Sigma, Saint Louis, USA) was performed by the microdilution method as described by Wiegand *et al.*[19].

### Virstatin effect on biofilms formed in static mode

*A. baumannii* biofilms were grown on 24-wells plates in Mueller Hinton (MH) broth with or without virstatin added at 20, 50 or 100 μM using DMSO as control. Plates were incubated for 24 h at 37°C without shaking. Attached cells were quantified by the protocol described by O’Toole and Kolter [20] or counted after their detachment by sonication. 25 μL were plated on MH agar and Colony Forming Units (CFU) were counted after 24 h of additional growth at 37°C. For testing the dispersing effect of virstatin, biofilms were grown for 24 h then challenged with increasing concentrations of virstatin (from 25 to 400 μM). Attached cells were similarly quantified after 24 h of additional growth.

All experiments were performed at least in triplicate. One way ANOVA was used to assess significant differences between a biofilm growth with and without virstatin. All data were statistically analyzed using Prism Graph Pad 5.

### Virstatin effect on biofilms formed in dynamic mode

The effect of virstatin on biofilms formed in dynamic mode was estimated with the BioFlux device (Fluxion Biosciences, South San Francisco, CA) as described by Benoit *et al.*[21] with some modifications. This system consists of a network of microfluidic laminar flow channels in which the growth of biofilms is controlled by shear force. The microfluidic channels (depth, 70 μm; width, 370 μm in PDMS : PolyDiMéthylSiloxane) were wetted with MH medium and inoculated with 10^7^ CFU.mL^-1^ of *A. baumannii* ATCC 17978 or of the other 39 strains. Following 1 h of incubation for cell attachment at 37°C, 1 mL fresh MH broth containing or not 100 μM virstatin was pumped at a flow rate of 0.3 dyn/cm^2^ from inlet wells through the channels to outlet wells. To obtain images, *A. baumannii* strains were grown for 24 h with a flow rate of 1 dyn/cm^2^. During biofilm formation, images were obtained using an inverted video-microscope (Leica DM IRBE), a digital camera (CoolSNAP Fx), and treated by Metamorph 7.0 software. Biofilm quantification was performed by image analysis using the Bioflux 200 software and the ‘area coverage’ module in ‘greyscale’ mode. The amount of sessile bacterial covering the flow cells was estimated in a defined and representative window, by an average value of coverage (μm^2^) depending on time (from 0-24 h). These values were then compared in absence or presence of 100 μM virstatin.

### Motility experiments

Motility of all *A. baumannii* strains was tested on polystyrene Petri dishes containing 10 g/L tryptone, 5 g/L NaCl with 0.3% agar (TSA; Difco) supplemented with 100 μM of virstatin or with the same volume of DMSO and incubated at 37°C overnight [22].

### Atomic force microscopy (AFM)

*A. baumannii* ATCC 17978 pellicles were transferred to collodion-coated glass slides after 8 h growth in MH broth supplemented or not with 100 μM of virstatin [11]. AFM imaging was performed as described in Marti *et al.*[11].

## Results

### Virstatin effect on *A. baumannii* growth in planktonic mode

We first examined the toxicity of virstatin on planktonically growing *A. baumannii* ATCC 17978 by generating growth curves with and without this compound and by counting CFU after addition of different concentrations of virstatin (Additional file 1: Figure S1-A&B). Neither virstatin nor its solvent (DMSO) inhibited *A. baumannii* growth at concentrations used in next steps of this study. The MIC of virstatin was finally measured at 1.6 mM.

### Virstatin effect on *A. baumannii* sessile bacteria

**Figure 1:**
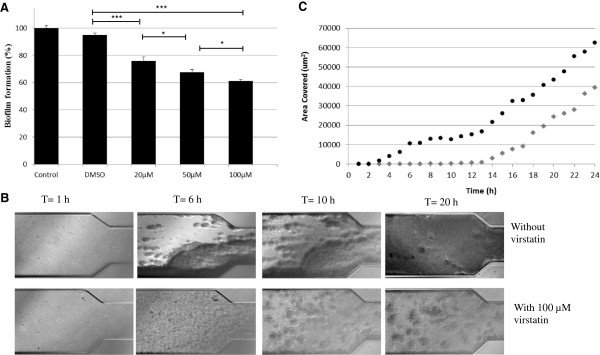
**Effect of virstatin on biofilms formed by *A. baumannii *. A**- Inhibition effect on *A. baumannii* ATCC 17978 biofilm development in static mode: percentages measured the 24 h-biofilm formation in presence of different concentrations of virstatin (and DMSO as control) compared to biofilm formation without virstatin. Experiments were performed in triplicate and results are presented as (mean ± standard error of mean). “***” for *P* < 0.0001 and “*” for *P* < 0.05, **B**- Effect on biofilm grown under flow shear (1 dyn/cm^2^) in Bioflux system: typical images of the microfluidic channels, representative of 3 independent assays, are shown. **C**- Kinetic of biofilm formation under flow shear in absence (●) or in presence (◆) of 100 μM virstatin. Analyse by the Bioflux 200 (Fluxion) software give an average value of coverage (μm^2^) depending on time (from 0-24 h).

To investigate the preventing effect of virstatin on biofilm growth in static mode, we measured *A. baumannii* ATCC 17978 biofilm production at varying virstatin concentrations. This quantitative determination was monitored by crystal violet colorimetric assay. Figure 1A shows clearly that virstatin exhibited an anti-biofilm activity, which was optimal for 100 μM with 38% biofilm formation inhibition. These data were confirmed by bacterial counting within the biofilms depending on virstatin concentrations (Additional file 1: Figure S1-B). We then examined the dispersing effect of virstatin on preformed biofilms. After growth for 24 h, biofilms were challenged by different concentrations of virstatin. Additional file 2: Figure S2 shows slight biofilm dispersion (12% for 400 μM) whatever the virstatin concentration. Therefore, virstatin exerted a marked inhibitory effect on *A. baumannii* biofilm formation but a weak dispersing effect on preformed biofilms.

We also investigated the effect of virstatin on biofilms formed in dynamic mode under flow shear, using the Bioflux microfluidic device. Figure 1B shows light micrographs taken from 0 to 24 h after the flow was initiated in the presence or absence of 100 μM virstatin. At 6 h, the micrographs showed a reduction of biofilm formation within the microfluidic channel irrigated by broth enriched in virstatin. After 20 h, in absence of virstatin, the channel was nearly filled by biofilm, whereas broth loaded with virstatin could still freely flow through the channel. The Figure 1C showed the kinetic of biofilm formation within the channels for both conditions. It clearly demonstrated that virstatin induced a time lag of nearly 10 hours for biofilm formation and that the biofilm production is reduced after 24 h. These data confirmed an antibiofilm action of virstatin within the first steps of biofilm formation.

### Virstatin effect on biofilms formed by clinical isolates

**Figure 2:**
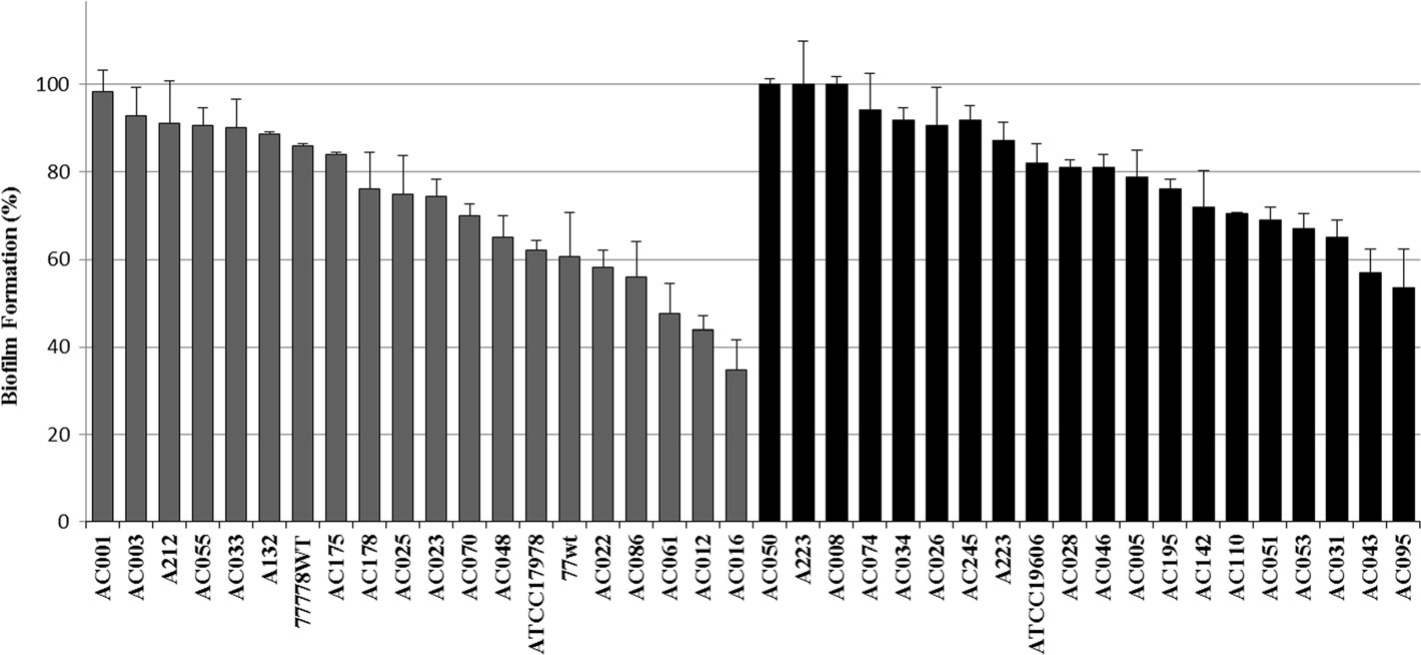
**Virstatin activity on biofilm formation by *A. baumannii *clinical isolates: Percentages measured the 24 h-biofilm formation in presence of 100 μM virstatin compared to biofilm formation without virstatin (but DMSO as control).** 100% denoted no activity of virstatin. *A. baumannii* strains forming biofilm on solid supports in black bars (■), strains forming pellicle in dark grey bars ().

To assess more broadly the anti-biofilm activity of virstatin, we tested this molecule on different *A. baumannii* clinical isolates as well as on the two reference strains. We examined a panel of 20 isolates forming pellicle and 20 other strains forming only biofilm on solid support [18]. As shown by Figure 2, 70% of clinical isolates presented at least a 10% decrease in their ability to form a biofilm. This decrease reached more than 65% for 100 μM virstatin. It should be noticed that the activity of virstatin was more marked on strains forming pellicle (75% of strains with a decrease from 10 to 65%) than on strains forming only biofilm on solid support (65% of strains with a decrease from 10 to 47%). The activity of virstatin was also examined on biofilms grown in dynamic mode. If all the tested strains were able to develop a biofilm in dynamic growth mode, virstatin could induce a time lag only for 60% of strains (Additional file 3: Table S1) and its activity was also more marked on strains forming pellicle (75%) than on strains forming biofilm on solid support (45%).

### Virstatin affects bacterial motility and pili production

**Figure 3:**
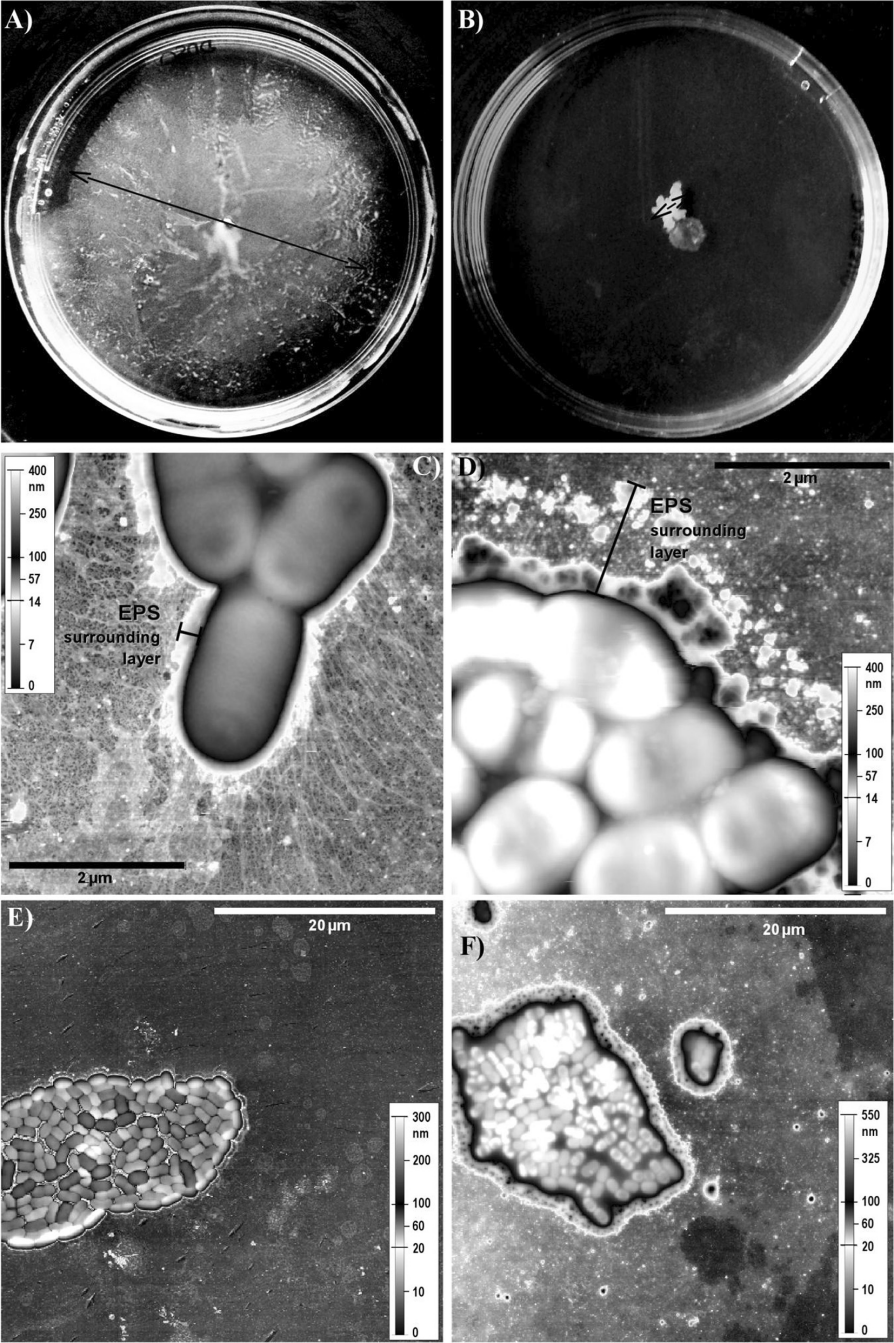
**Virstatin affects bacterial motility and pili production. A&B**- Plates of motility of *A. baumannii* ATCC 17978 strain in presence **(B)** or absence **(A)** of virstatin in semi-solid 0.3% TSA after 24 h growth at 37°C. Typical plates are shown. The diameter of the motility zone decreased when medium is supplemented with 100 μM virstatin. The experiments were performed in triplicate. **C-F** - AFM images of *A. baumannii* ATCC 17978 water-facing side pellicles after 8 h growth: **C&E** without virstatin; D&F with 100 μM virstatin. Topographic representation using an uncommon scale made to emphasize the bacterial pili and the ‘EPS’ (extrapolymeric substances) surrounding layer.

As virstatin may affect pili production, we performed motility assays. 0.3% TSA plates containing or not 100 μM virstatin were inoculated by *A. baumannii* ATCC 17978 [12]. Figure 3A & B shows that virstatin significantly inhibited bacterial migration. This motility assay was performed for all the strains (Additional file 3: Table S1) and the data showed that over the 30 strains that were mobile, 60% underwent a decrease of their motility.

In order to check the presence of pili after virstatin action, the pellicles of *A. baumannii* ATCC 17978 were examined by AFM. In absence of virstatin, observation of the water-facing side of the pellicles revealed the significant presence of pili (0.5 to 2 μm in length) around bacteria, which were located on the border of the colonies (Figure 3C). In contrast when bacteria grew with virstatin, the amount of pili drastically decreased (Figure 3D). Moreover, we observed a layer surrounding bacteria, the average height and width of which were greater in presence (height: 100 nm, width: 1–1.5 μm) than in absence of virstatin (height: 50 nm, width: 0.1 μm, Figure 3C & D). This was most likely extrapolymeric substances (EPS) overproduced in presence of virstatin. At a lower resolution, images showed strong differences between both pellicles (Figure 3E & F). The pellicle formed in absence of virstatin was quite dense and was formed of a single bacterial layer, giving a colony mean height of (0.20 ± 0.05) μm. In presence of virstatin, the pellicle appeared less dense and formed with more than one layer of bacteria. In this case, the colony was about (0.50 ± 0.05) μm in height.

## Discussion

Targeting essential protein-protein interactions in bacterial function is a very promising but challenging therapeutic approach which is increasingly developed [14,15]. In this context, small organic molecules (ring fused 2-pyridones) were tested as pilicide or curlicide on uropathogenic *Escherichia coli* (UPEC) with success. In targeting either type 1 pili or amyloid fibers biogenesis, these compounds can prevent biofilm formation and attenuate UPEC virulence [14]. Virstatin is similarly used to decrease the expression of T4P which are virulence factors in *V. cholerae*[15,16].

In this study, we demonstrated that virstatin presents also an interesting antibiofilm activity as it could reduce by 65% the biofilm production in some *A. baumannii* clinical isolates and is active on more than 70% of the tested strains. Virstatin remained also active against biofilms formed under flow shear inducing clearly a delay of the development of this type of biofilm. These results are in favor of its action on pili biogenesis. Indeed, these cell surface appendages are known to be involved in the first stages of biofilm development promoting, initial adhesion and surface colonization but also microcolonies formation [8]. Thus the decrease of virstatin activity at a later phase of biofilm development (48 h) is not surprising, as pili expression may not be required. Moreover, the AFM observations confirmed the hypothesis suggesting an inhibitory effect of virstatin on the biogenesis of pili: they pointed out a drastic under-production of these appendages in the presence of the molecule (Figure 3C & D). Finally, motility experiments demonstrated that virstatin clearly affected the migration of *A. baumannii* on semi-solid surface. This motility has been examined by Clemmer and colleagues [12] who showed that the loss of *pilT*, a gene involved in twitching mediated by T4P, resulted in a 54% reduction in motility of non-flagellar *A. baumannii*. In addition, AFM micrographs showed the overexpression of EPS in biofilms grown with virstatin. Recently Wang *et al*. [23] demonstrated that if deletion of T4P in *Pseudomonas aeruginosa* resulted in a reduction of biofilm biomass in flow cell and in pellicles, it concomitantly resulted in an overproduction of the Psl polysaccharide in the microcolonies. Overall, these results are in favor of an action of virstatin on T4P pili biogenesis.

## Conclusions

The emergence of *A. baumannii* as one of the most problematic nosocomial pathogen has made necessary the development of new strategies to impair its ability to persist in hospital environment. Here, we have tested a strategy based on the prevention of the pili biosynthesis to reduce the *A. baumannii* biofilm production. This was successful with the use of a small organic compound, the virstatin, which demonstrated an activity on 70% of the clinical isolates with a decrease in biofilm production that could reach 65%.

## Competing interests

The authors declare that they have no competing interests.

## Authors’ contributions

YN-C, TJ, ED conceived and designed the experiments. YN-C, MB-M, MP-C, SM, SA, MN performed the experiments. YN-C, SA, TJ, JV, ED analyzed the data. MP-C, SM, SA, JV contributed to materials/technical support. ED wrote the paper. All authors read and approved the final manuscript.

## Supplementary Material

Additional file 1: Figure S1 Virstatin effect on *A. baumannii* ATCC 17978 growth in planktonic and biofilm modes. A- Growth curves of *A. baumannii* in MH broth (☐), MH broth with 0.5% DMSO (volume used for the addition of 100 μM virstatin) (◇), or MH broth with 100 μM virstatin (△). B- *A. baumannii* colony forming units after a 24 h planktonic (◆) and biofilm (●) growth depending on virstatin concentrations. Curves are given as an average of 3 replicates. Click here for file

Additional file 2: Figure S2 Dispersing effect of virstatin. Virstatin added at 100 μM on 24 h preformed biofilms. Quantification of biofilm biomass was made by crystal violet staining after additional 24 h growth. “*” for *P* < 0.05. Click here for file

Additional file 3: Table S1 Virstatin effect on *A. baumannii* clinical isolates. Click here for file

## References

[B1] Dijkshoorn L Nemec A Seifert H An increasing threat in hospitals: multidrug-resistant *Acinetobacter baumannii* Nat Rev Microbiol 2007 5 12 939 951 10.1038/nrmicro1789 18007677

[B2] Rodriguez-Bano J Marti S Soto S Fernandez-Cuenca F Cisneros JM Pachon J Pascual A Martinez-Martinez L McQueary C Actis LA Vila J Spanish Group for the Study of Nosocomial Infections (GEIH) Biofilm formation in *Acinetobacter baumannii*: associated features and clinical implications Clin Microbiol Infec 2008 14 3 276 278 10.1111/j.1469-0691.2007.01916.x 18190568

[B3] Giannouli M Antunes LC Marchetti V Triassi M Visca P Zarrilli R Virulence-related traits of epidemic *Acinetobacter baumannii* strains belonging to the international clonal lineages I-III and to the emerging genotypes ST25 and ST78 BMC Infect Dis 2013 13 282 10.1186/1471-2334-13-282 23786621 PMC3691691

[B4] Bentancor LV O’Malley JM Bozkurt-Guzel C Pier GB Maira-Litran T Poly-N-acetyl-beta-(1–6)-glucosamine is a target for protective immunity against *Acinetobacter baumannii* infections Infect Immun 2012 80 2 651 656 10.1128/IAI.05653-11 22104104 PMC3264292

[B5] Choi AH Slamti L Avci FY Pier GB Maira-Litran T The pgaABCD locus of *Acinetobacter baumannii* encodes the production of poly-beta-1-6-N-acetylglucosamine, which is critical for biofilm formation J Bacteriol 2009 191 19 5953 5963 10.1128/JB.00647-09 19633088 PMC2747904

[B6] Gaddy JA Actis LA Regulation of *Acinetobacter baumannii* biofilm formation Future Microbiol 2009 4 3 273 278 10.2217/fmb.09.5 19327114 PMC2724675

[B7] Iwashkiw JA Seper A Weber BS Scott NE Vinogradov E Stratilo C Reiz B Cordwell SJ Whittal R Schild S Feldman MF Identification of a general O-linked protein glycosylation system in *Acinetobacter baumannii* and its role in virulence and biofilm formation PLoS Pathog 2012 8 6 e1002758 10.1371/journal.ppat.1002758 22685409 PMC3369928

[B8] Mikkelsen H Sivaneson M Filloux A Key two-component regulatory systems that control biofilm formation in *Pseudomonas aeruginosa* Environ Microbiol 2011 13 7 1666 1681 10.1111/j.1462-2920.2011.02495.x 21554516

[B9] Tomaras AP Dorsey CW Edelmann RE Actis LA Attachment to and biofilm formation on abiotic surfaces by *Acinetobacter baumannii*: involvement of a novel chaperone-usher pili assembly system Microbiology 2003 149 Pt 12 3473 3484 14663080 10.1099/mic.0.26541-0

[B10] Di Nocera PP Rocco F Giannouli M Triassi M Zarrilli R Genome organization of epidemic *Acinetobacter baumannii* strains BMC Microbiol 2011 11 224 10.1186/1471-2180-11-224 21985032 PMC3224125

[B11] Marti S Nait Chabane Y Alexandre S Coquet L Vila J Jouenne T De E Growth of *Acinetobacter baumannii* in pellicle enhanced the expression of potential virulence factors PloS one 2011 6 10 e26030 10.1371/journal.pone.0026030 22046254 PMC3203104

[B12] Clemmer KM Bonomo RA Rather PN Genetic analysis of surface motility in *Acinetobacter baumannii* Microbiology 2011 157 Pt 9 2534 2544 21700662 10.1099/mic.0.049791-0 PMC3352170

[B13] Klausen M Heydorn A Ragas P Lambertsen L Aaes-Jorgensen A Molin S Tolker-Nielsen T Biofilm formation by *Pseudomonas aeruginosa* wild type, flagella and type IV pili mutants Mol Microbiol 2003 48 6 1511 1524 10.1046/j.1365-2958.2003.03525.x 12791135

[B14] Cegelski L Pinkner JS Hammer ND Cusumano CK Hung CS Chorell E Aberg V Walker JN Seed PC Almqvist F Chapman MR Hultgren SJ Small-molecule inhibitors target *Escherichia coli* amyloid biogenesis and biofilm formation Nat Chem Biol 2009 5 12 913 919 10.1038/nchembio.242 19915538 PMC2838449

[B15] Hung DT Shakhnovich EA Pierson E Mekalanos JJ Small-molecule inhibitor of *Vibrio cholerae* virulence and intestinal colonization Science 2005 310 5748 670 674 10.1126/science.1116739 16223984

[B16] Shakhnovich EA Hung DT Pierson E Lee K Mekalanos JJ Virstatin inhibits dimerization of the transcriptional activator ToxT Proc Natl Acad Sci USA 2007 104 7 2372 2377 10.1073/pnas.0611643104 17283330 PMC1892951

[B17] Magiorakos AP Srinivasan A Carey RB Carmeli Y Falagas ME Giske CG Harbarth S Hindler JF Kahlmeter G Olsson-Liljequist B Paterson DL Rice LB Stelling J Struelens MJ Vatopoulos A Weber JT Monnet DL Multidrug-resistant, extensively drug-resistant and pandrug-resistant bacteria: an international expert proposal for interim standard definitions for acquired resistance Clin Microbiol Infec 2012 18 3 268 281 10.1111/j.1469-0691.2011.03570.x 21793988

[B18] Marti S Rodriguez-Bano J Catel-Ferreira M Jouenne T Vila J Seifert H De E Biofilm formation at the solid–liquid and air-liquid interfaces by *Acinetobacter* species BMC Res Notes 2011 4 5 10.1186/1756-0500-4-5 21223561 PMC3023692

[B19] Wiegand I Hilpert K Hancock RE Agar and broth dilution methods to determine the minimal inhibitory concentration (MIC) of antimicrobial substances Nat Protoc 2008 3 2 163 175 10.1038/nprot.2007.521 18274517

[B20] O’Toole GA Kolter R Initiation of biofilm formation in *Pseudomonas fluorescens* WCS365 proceeds via multiple, convergent signalling pathways: a genetic analysis Mol Microbiol 1998 28 3 449 461 10.1046/j.1365-2958.1998.00797.x 9632250

[B21] Benoit MR Conant CG Ionescu-Zanetti C Schwartz M Matin A New device for high-throughput viability screening of flow biofilms Appl Environ Microbiol 2010 76 13 4136 4142 10.1128/AEM.03065-09 20435763 PMC2897429

[B22] McQueary CN Actis LA *Acinetobacter baumannii* biofilms: variations among strains and correlations with other cell properties J Microbiol 2011 49 2 243 250 10.1007/s12275-011-0343-7 21538245

[B23] Wang S Parsek MR Wozniak DJ Ma LZ A spider web strategy of type IV pili-mediated migration to build a fibre-like Psl polysaccharide matrix in *Pseudomonas aeruginosa* biofilms Environ Microbiol 2013 15 8 2238 2253 10.1111/1462-2920.12095 23425591 PMC4466117

