# Supplementary material for: Virstatin inhibits biofilm formation and motility of Acinetobacter baumannii
Source: BMC Microbiol. 2014 Mar 12;14:62. doi: 10.1186/1471-2180-14-62 (PMC4007623; doi:10.1186/1471-2180-14-62)
Supplement: Additional file 1: Figure S1 — Virstatin effect on A. baumannii ATCC 17978 growth in planktonic and biofilm modes. A- Growth curves of A. baumannii in MH broth (☐), MH broth with 0.5% DMSO (volume used for the addition of 100 μM virstatin) (◇), or MH broth with 100 μM virstatin (△). B- A. baumannii colony forming units after a 24 h planktonic (◆) and biofilm (●) growth depending on virstatin concentrations. Curves are given as an average of 3 replicates. [file 1471-2180-14-62-S1.doc]

**Additional File 1**

**Figure S1.** **Virstatin effect on *A. baumannii* ATCC 17978 growth in planktonic and biofilm modes. A- Growth curves of *A. baumannii*** in MH broth (🞏), MH broth with 0.5% DMSO (volume used for the addition of 100 µM virstatin) (◇), or MH broth with 100 µM virstatin (△). **B- Numbers of CFU/mL after a 24h planktonic (◆) and biofilm (●) growth depending on virstatin concentrations.** Curves are given as an average of 3 replicates.

**
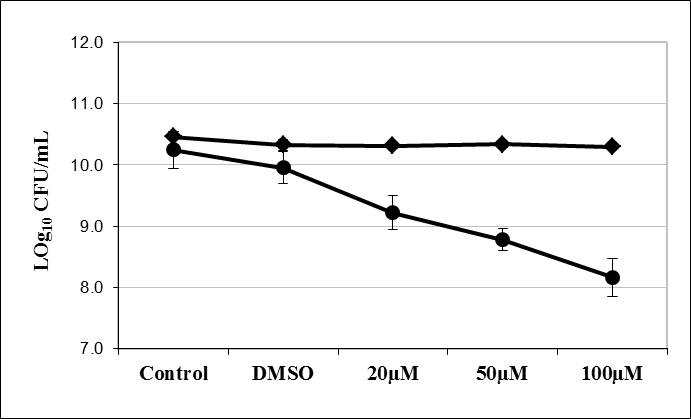

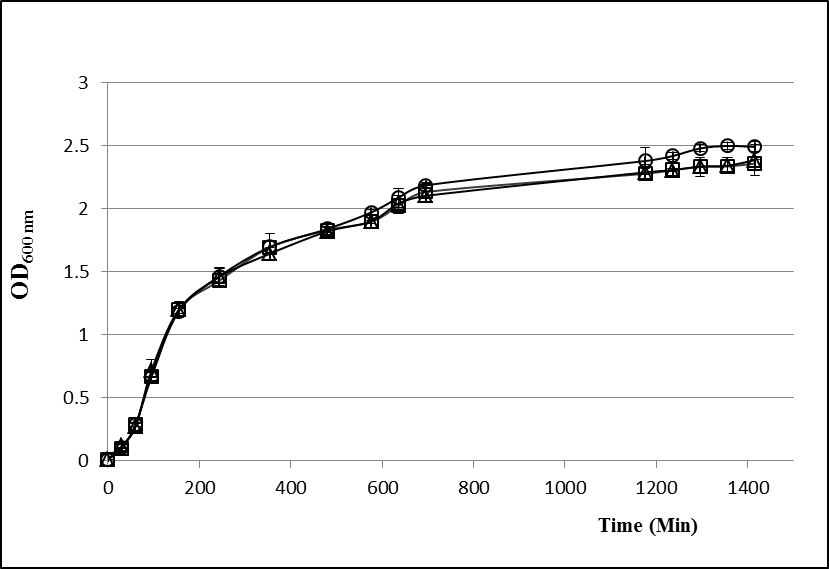
**
