# Supplementary material for: Virstatin inhibits biofilm formation and motility of Acinetobacter baumannii
Source: BMC Microbiol. 2014 Mar 12;14:62. doi: 10.1186/1471-2180-14-62 (PMC4007623; doi:10.1186/1471-2180-14-62)
Supplement: Additional file 2: Figure S2 — Dispersing effect of virstatin. Virstatin added at 100 μM on 24 h preformed biofilms. Quantification of biofilm biomass was made by crystal violet staining after additional 24 h growth. “*” for P < 0.05. [file 1471-2180-14-62-S2.docx]

**Additional File 2**

**Figure S2. Dispersing effect of virstatin.** Virstatin added at 100 µM on 24h preformed biofilms. Quantification of biofilm biomass made by crystal violet staining after an additional 24h growth. “*” for *P*<0.05


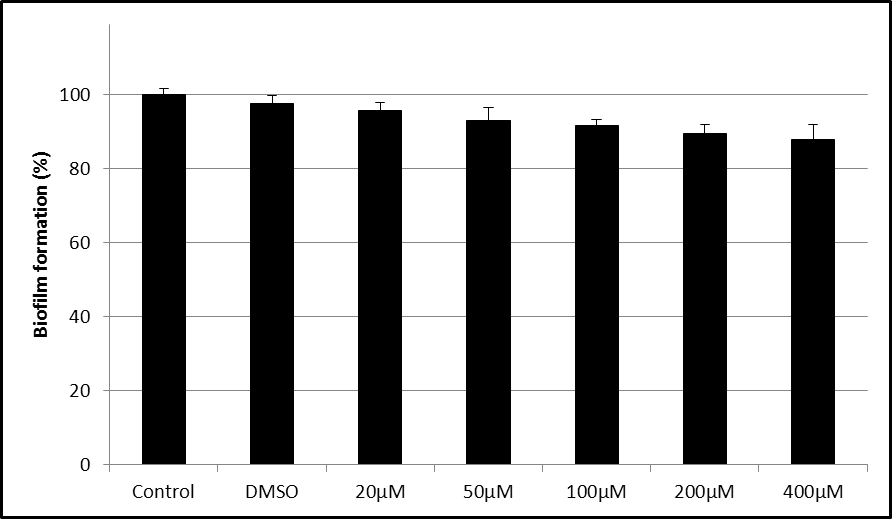


*

*
