# Supplementary material for: Virstatin inhibits biofilm formation and motility of Acinetobacter baumannii
Source: BMC Microbiol. 2014 Mar 12;14:62. doi: 10.1186/1471-2180-14-62 (PMC4007623; doi:10.1186/1471-2180-14-62)
Supplement: Additional file 3: Table S1 — Virstatin effect on A. baumannii clinical isolates. [file 1471-2180-14-62-S3.docx]

|  |  |  |  |  | **Motility,** |
| --- | --- | --- | --- | --- | --- |
|  |  |  |  | **Time-lag (h) in** | **Ø without/with** |
| **Strains** | **Isolate source** | **MDR/XDR^(◆)^** | **Static biofilm** | **dynamic biofilm** | **virstatin** |
| **Pellicle +** |  |  |  |  |  |
| AC055 | Exudate | MDR | - | - | 3.4±0.5/2.5±0.8 (*) |
| AC001 | Respiratory | XDR | - | 6 | 2.1±0.5/1.0±0.1 (**) |
| AC172 | Urine | S | 2 | 3 | 1.9±0.1/0.5±0.2 (**) |
| AC003 | Blood | S | 7 | 4 | - |
| A212 | Environment | S | 9 | - | NM |
| AC033 | Urine | S | 10 | 2 | - |
| A132 | Urine | S | 12 | - | - |
| 77778wt | ND | ND | 14 | 8 | NM |
| AC175 | Exudate | XDR | 16 | 4 | NM |
| AC178 | Exudate | MDR | 21 | 4 | 2.7±0.6/1.0±0.3 (**) |
| AC048 | ND | S | 23 | 5 | 2.0±0.6/0.9±0.2 (**) |
| AC025 | Respiratory | S | 25 | 9 | 3.8±0.1/1.0±0.2 (**) |
| AC023 | Exudate | MDR | 26 | - | NM |
| ATCC 17978 | Blood culture | S | 35 | 10 | 5.4±0.2/3.3±0.2 (**) |
| AC022 | Abcess | XDR | 40 | 9 | 1.8±0.6/0.4±0.0 (**) |
| 77WT | Urine | S | 40 | 12 | NM |
| AC086 | ND | XDR | 41 | - | 3.8±0.8/1.3±0.1 (**) |
| AC061 | Urine | MDR | 47 | 2 | 2.9±0.3/1.0±0.3 (**) |
| AC012 | Exudate | MDR | 53 | 3 | 3.6±0.1/2.6±0.7 (*) |
| AC016 | Urine | MDR | 65 | 4 | NM |
|  |  |  |  |  |  |
| **Biofilm +** |  |  |  |  |  |
| AC050 | Urine | MDR | - | - | - |
| A223 | Exudate | XDR | - | - | - |
| AC008 | Respiratory | S | - | - | - |
| AC074 | Respiratory | XDR | 6 | 4 | 3.5±0.5/2.2±0.6 (*) |
| AC034 | Urine | MDR | 8 | 1 | - |
| AC245 | Blood | S | 8 | - | - |
| AC051 | Urine | MDR | 8 | - | NM |
| AC026 | Urine | XDR | 14 | 12 | - |
| AC028 | ND | S | 19 | 3 | 3.0±0.4/2.2±0.1 (*) |
| AC046 | Urine | S | 19 | - | 1.8±0.6/0.5±0.1 (**) |
| AC005 | Respiratory | MDR | 21 | - | 2.4±0.8/1.1±0.1 (**) |
| ATCC19606 | Urine | S | 22 | 1 | NM |
| AC195 | ND | MDR | 24 | - | 2.0±0.2/1.1±0.3 (**) |
| AC142 | Respiratory | XDR | 28 | - | 1.1±0.2/0.4±0.0 (**) |
| AC110 | ND | S | 30 | ND | - |
| AC070 | Blood | S | 30 | 1 | - |
| AC053 | ND | S | 33 | - | NM |
| AC031 | Exudate | MDR | 35 | 2 | 2.8±0.6/1.2±0.6 (**) |
| AC043 | Urine | XDR | 43 | 2 | NM |
| AC095 | ND | S | 47 | 3 | 4.6±0.4/2.7±0.1 (**) |

**Table S1. Virstatin effect on *A. baumannii* clinical isolates**

(-) denotes no activity; (*) 10-40% of motility reduction; (**) 40-80% of motility reduction; “NM” for non-motile; “ND” for not determined; S for sensitive and ^(^**^◆)^** MDR for Multidrug-resistant, XDR extensively drug-resistant as defined by Magiorakos *et al.,* [20].
